# A promising first year: Happy birthday AE&M!

**DOI:** 10.1590/2359-3997000000163

**Published:** 2016-01-01

**Authors:** Marcello D. Bronstein

**Affiliations:** 1 Hospital das Clínicas Faculdade de Medicina Universidade de São Paulo São Paulo SP Brasil Editor-chefe da Archives of Endocrinology and Metabolism, chefe da Unidade de Neuroendocrinologia, Serviço de Endocrinologia e Metabologia, Hospital das Clínicas, Faculdade de Medicina da Universidade de São Paulo (HCFMUSP), São Paulo, SP, Brasil

The *Archives of Endocrinology and Metabolism* (AE&M) are completing its first year. Despite its name in English and the mandatory use of this language for all articles, the former “*Arquivos Brasileiros de Endocrinologia e Metabologia*” remains the official *Journal of the Brazilian Society of Endocrinology and Metabolism*. I believe that such change will progressively increase the knowledge and interest of endocrinologists around the word on our journal, which, without losing its Brazilian origin and identity, will become more and more an international publication. Consequently its impact factor, unavailable during two years due to its name change, is expected to augment, leading to the increase of submissions of good original basic, translational and clinical research, in a healthy vicious cycle. In order to pursue the increase of the impact factor, original and review papers should be privileged, in detriment of case reports. As a matter of fact, many medical journals increased its impact factor restricting the acceptance of case reports. On the other hand, many journals are dealing specifically with this kind of medical article.

During 2015, 416 manuscripts have been submitted to AE&M. From them, 71.4% were original research, and the remainder consisted of case reports (18%), review articles (5.5%), brief reports (4.6%), letter to the editor (0.2%), and consensus statement (0.2%). From the total of submitted manuscripts, 306 came to a decision, and 79 have been accepted for publication (26%).

Regarding the number of manuscripts per country, Brazil submitted 146, Turkey 61, China 16, India 11, Iran 10, Argentina 9, Portugal 8, Italy 7, Romania 5, and Egypt 4. Additionally, the following countries also submitted articles: Algeria, Bulgaria, Chile, Colombia, Croatia, Cyprus, Greece, Hungary, Mexico, Nigeria, Pakistan, Poland, Saudi Arabia, Serbia, Slovakia, Spain, Taiwan, Tunisia, USA, and Venezuela.

In this second year of my task as Editor-in-Chief of the AE&M, I intend to strengthen the relationship with colleagues of other countries, asking for reviews and mini-reviews. For this purpose I expect to count on the international editorial board of our journal.

Last but not least, I would like to acknowledge and warmly thank all my Associate Editors for their outstanding collaboration, as well as the Brazilian and International reviewers for their time and expertise, selecting manuscripts which enriched the quality of our AE&M.

